# Supplementary material for: Content-rich biological network constructed by mining PubMed abstracts
Source: BMC Bioinformatics. 2004 Oct 8;5:147. doi: 10.1186/1471-2105-5-147 (PMC528731; doi:10.1186/1471-2105-5-147)
Supplement: Additional File 2 — The original results of the above study (non-essential files are deleted to keep the file size under the limit set by BMC bioinformatics). [file 1471-2105-5-147-S2.bz2 › chilibotAdditionalFile2/dip20/758ID11500384E18144/html/DNAA_REPA.html]

 


 **DNAA** and **REPA** 
  
Found 61 abstracts in PubMed, retrieved 20.  
 

 What does Google say? 
 PDF only 
| .edu only 

---

**Interactive relationship** (e.g. stimulation, inhibition, etc)

**Neutral relationship**- Thus, the result shows that physical tethering, either non covalent or covalent, of domain I and IV of  **DnaA**  and interaction of both domains with  **RepA** , are critical for replication initiation.  Ref: 11500384 EMBO J, 2001
- Neither domain I nor IV of  **DnaA**  could individually collaborate with  **RepA**  to promote pSC101 replication.  Ref: 11500384 EMBO J, 2001
- The N terminal, but not the C terminal, region of  **RepA**  interacted with  **DnaA**  in vitro.  Ref: 11500384 EMBO J, 2001
- None of these mutations drastically changed the structure or thermal stability of the wild type  **RepA**  protein, but in all cases an enhanced interaction with host encoded  **DnaA**  protein was detected by gel filtration chromatography.  Ref: 12562807 J Bacteriol, 2003
- We propose possible explanations for the host range effect of the different  **repA**  mutants, including the enhancement of limiting interactions of  **RepA**  with specific host replication factors such as  **DnaA** .  Ref: 12562807 J Bacteriol, 2003
- Mechanistic aspects of  **DnaA**   **RepA**  interaction as revealed by yeast forward and reverse two hybrid analysis.  Ref: 11500384 EMBO J, 2001
- Using yeast forward and reverse two hybrid analysis and biochemical techniques, we present novel and definitive in vivo and in vitro evidence that both the N terminal domain I and C terminal domain IV of the host encoded  **DnaA**  initiator protein of Escherichia coli interact physically with plasmid encoded  **RepA**  initiator of pSC101.  Ref: 11500384 EMBO J, 2001
- Binding of  **RepA**  to the  **RepA**  boxes was ordered and sequential, with the box closest to the  **DnaA**  binding site box 1 occupied first and the most distant boxes boxes 3 and 4 occupied last.  Ref: 12644491 J Bacteriol, 2003
- The iteron like region was identified upstream of the  **repA**  gene and consisted of two clusters of repeated sequences 17 bp long separated by a putative  **DnaA**  box.  Ref: 11407917 Plasmid, 2001
- The initiation of replication from oriV RSF1010, the replication origin of the broad host range plasmid RSF1010, depends on  **RepA**  helicase, RepB primase, and RepC initiator protein, encoded by RSF1010 itself, while this initiation event in E. coli is independent of  **dnaA** , dnaB, dnaC, and dnaG Scherzinger et al.  Ref: 8690079 FEBS Lett, 1996

**Non-interactive relationship** (e.g. studied together, co-existance, homology, etc.)

- Additionally, they provide further evidence that the origin region  **RepA**   **DnaA**  DNA complex initiating replication of pSC101 also mediates the partitioning of pSC101 plasmids at cell division.  Ref: 7665462 J Bacteriol, 1995
- **RepA**  protein of pPS10 promotes in vitro plasmid replication in extracts from E. coli, and this replication depends on host proteins  **DnaA** , DnaB, DnaG, and SSB.  Ref: 7635822 J Bacteriol, 1995
- This oriV RsF1010  **dnaA**  box could not replicate in a dnaA46 strain in which only  **RepA**  and RepC were supplied, even at a permissive temperature.  Ref: 8690079 FEBS Lett, 1996
- Although, interaction of  **DnaA**  with DnaB could not substitute for  **RepA**  DnaB interaction for helicase loading,  **DnaA**  along with integration host factor, DnaC, and  **RepA**  was essential for helicase loading.  Ref: 9874774 Proc Natl Acad Sci U S A, 1999
- The  **DnaA**  replication initiation protein  [ **REPA** ]  has been shown to be essential for DNA strand opening at the AT rich region of the replication origin of the Escherichia coli chromosome as well as serving to recruit and position the DnaB replicative helicase at this open region.  Ref: 12835421 Proc Natl Acad Sci U S A< MedlineTA>Proc Natl Acad Sci U S A, 1999
- To our knowledge, this work reports the first chromosome mutant described in the literature that allows the host range broadening of a plasmid, highlights the essential role played by  **DnaA**  in the establishment of pPS10 replicon in E. coli and provides support for the hypothesis that interactions between  **RepA**  and  **DnaA**  modulate the establishment of pPS10 in that bacteria and probably in other species.  Ref: 12366845 Mol Microbiol, 2002
- Although many bacterial chromosomes require only one replication initiator protein, e.g.,  **DnaA** , most plasmid replicons depend on dual initiators host encoded  **DnaA**  and plasmid encoded Rep  [ **REPA** ]  initiator protein for replication initiation.  Ref: 9874774 Proc Natl Acad Sci U S A, 1999
- Missense mutations in the plasmid encoded  **RepA**  protein or mutation of the Escherichia coli topoisomerase I gene enable autonomous replication of a 310 bp pSC101 DNA fragment that contains only the actual replication origin plus binding sites for  **RepA**  and the host encoded  **DnaA**  protein.  Ref: 7665462 J Bacteriol, 1995
- Using reactivity to KMnO4 in vivo as an assay, we find that, like initiation, origin opening of the Escherichia coli plasmid P1 requires the host initiators  **DnaA**  and HU and the plasmid encoded initiator  **RepA** .  Ref: 9733797 J Biol Chem, 1998
- We showed in this work that a newly constructed origin consisting of an oriV RSF1010 and a  **DnaA**  protein binding site, the  **dnaA**  box, inserted near oriV RSF1010 oriV RSF1010  **dnaA**  box could function without RepB primase, but required  **RepA**  and RepC.  Ref: 8690079 FEBS Lett, 1996
- The concentrations of  **RepA**  and  **DnaA** , which interact with each other and with plasmid DNA in the origin region T.  Ref: 7665462 J Bacteriol, 1995
- The order of the genes with established functions was groELS trmE rnpA rpmH  **dnaA**  dnaN gyrB atpCDGAH FEB gidA fdx hscA hscB nifS ilvDC rep  [ **REPA** ]  trxA rho.  Ref: 9516544 Curr Microbiol, 1998
- Moreover, neither replicon requires the host  **DnaA** , DnaG, and Rep  [ **REPA** ]  functions.  Ref: 10438819 J Virol, 1999
